# Affective Instability in Daily Life Is Predicted by Resting Heart Rate Variability

**DOI:** 10.1371/journal.pone.0081536

**Published:** 2013-11-29

**Authors:** Peter Koval, Barbara Ogrinz, Peter Kuppens, Omer Van den Bergh, Francis Tuerlinckx, Stefan Sütterlin

**Affiliations:** 1 Faculty of Psychology and Educational Sciences, KU Leuven, Leuven, Belgium; 2 Department of Psychology, Lillehammer University College, Lillehammer, Norway; 3 Department of Neuropsychiatry and Psychosomatic Medicine, Oslo University Hospital Rikshospitalet, Oslo, Norway; University of Sao Paulo, Brazil

## Abstract

Previous research has shown that being affectively unstable is an indicator of several forms of psychological maladjustment. However, little is known about the mechanisms underlying affective instability. Our research aims to examine the possibility that being prone to extreme fluctuations in one’s feelings is related to maladaptive emotion regulation. We investigated this hypothesis by relating affective instability, assessed in daily life using the experience sampling method, to self-reported emotion regulation strategies and to parasympathetically mediated heart rate variability (HRV), a physiological indicator of emotion regulation capacity. Results showed that HRV was negatively related to instability of positive affect (as measured by mean square successive differences), indicating that individuals with lower parasympathetic tone are emotionally less stable, particularly for positive affect.

## Introduction

Although everyone’s feelings change, some individuals are more affectively labile than others and experience larger and more frequent changes in affect. Extreme affective instability is not without harm, however. It appears as a DSM criterion for several psychiatric disorders [1] and has been related to several forms of psychological maladjustment [2]. However, little is known about the factors associated with affective instability. What characterizes a person who is more emotionally unstable than another? In this paper we examine the possibility that being prone to large fluctuations in one’s feelings is related to self-reported use of emotion regulation (reappraisal and suppression) as well as to a physiological measure of emotion regulation capacity, parasympathetically mediated heart rate variability (HRV).

### Affective instability

Affective instability, also referred to as “emotional lability”, is generally conceptualized as a pattern of frequent and large mood shifts over time [3]. While emotions that are resistant to change may indicate psychological ill-health [4-6], research has also shown that high levels of affective instability are related to maladaptive outcomes. In this domain, affective instability has been operationalized in different ways. A number of studies have examined the within-person standard deviation of affect over time, showing that greater affective variability in negative affect is related to poor psychological health [7], neuroticism [8-10] and depression [11,12]. Greater affective variability in positive affect has also been associated with neuroticism [9,13], depression [11], borderline symptomatology [3,14] and low self-esteem [15,16]. 

However, a within-person standard deviation does not necessarily reflect large and frequent shifts, but merely the range of a person’s affect levels with no consideration of the order in which changes occur [2,3]. Therefore, more recently, the mean square successive difference (MSSD) has been proposed as a measure of affective instability [17]. The MSSD reflects the extent to which consecutively measured moods differ from each other and therefore incorporates both the size of affective changes as well as their temporal order [17]. This temporally sensitive index of affective instability is also related to psychological ill health. For instance, greater instability of negative affect is found in borderline personality and bipolar patients as compared to healthy controls and patients suffering from other mental diseases [3,18,19] and is hypothesized to play a role in several other mood disorders [2]. Among adolescents, instability of positive and negative affect has also been associated with symptoms of anxiety and depression as well as with aggressive behavior [20]. 

As a result, some investigators have argued that greater affective instability reflects “dysregulation of affect” [21,22]. While affect regulation can in principle be in any direction, people most often employ it to down-regulate overly intense experiences [10]. When such regulatory efforts are ineffective, an individual will be less able to control large affective shifts and fluctuations, which could result in higher levels of instability. Indeed, the affect dysregulation assumed to underlie the abovementioned disorders is thought to give rise to larger mood swings and reactivity, which in turn may manifest themselves as increased affective instability [2,23]. However, impaired or ineffective emotion regulation is not only a feature of psychopathology, but is also associated with normal variation in personality and well-being [24]. Thus, an important first step is to investigate how emotion regulation relates to affective instability in a non-clinical sample. While there is ample evidence supporting the notion that deficiencies in emotion regulation are related to altered emotional responding [10,25-27], less is known about how emotion regulation is directly associated with affective instability. Our goal in the present study was to address this fundamental question.

### Emotion regulation

Emotion regulation refers to all processes – automatic or controlled, conscious or unconscious – that may increase, maintain or decrease a person’s emotional state [28]. Emotion regulation is a multifaceted concept that can be indexed by both subjective and objective measures. On a subjective level, people can be asked to report which strategies they use to regulate their emotions. A prominent example is the framework developed by Gross and John [24] that distinguishes between several emotion regulation strategies, of which reappraisal and suppression are the most widely studied. On an objective level, besides behavioral indicators of emotion regulation [29], recent research has suggested a biological marker of emotion regulation capacity, namely heart rate variability [25]. 

#### Self-reported emotion regulation

Reappraisal is defined as a cognitive reevaluation of the emotion-eliciting situation in order to modify its impact [24]. Suppression consists of inhibiting the behavioral expression of one’s emotions [24]. 

In terms of their consequences for experienced affect, reappraisal and suppression seem to be rather different. By and large, reappraisal is associated with beneficial consequences, such as lower levels of negative emotions and higher levels of positive emotions, well-being, and social relationship quality [24,30,31]. Suppression, on the other hand, by definition decreases the extent to which emotions are overtly expressed, but this does not seem to come with an associated change in experienced affect. Indeed, research has shown that when people suppress the expression of their emotions, their affective experience remains relatively unaltered. If anything, suppression seems to enhance negative emotions [24,32] and stress [33,34]. 

Consequently, with the goal of reappraisal being to lessen the emotional impact of a situation, we expect that it will be associated with lower instability of both positive and negative affect (as indexed by lower MSSD). As suppression seems to primarily affect the outward expression and less so the experienced intensity of emotion, we do not have strong expectations about it being related to affective instability in terms of experienced affect. 

#### Heart rate variability

Resting HRV refers to the beat-to-beat variation in heart rate. Specific parameters referring to the specific changes in heart rate [35,36] indicate the extent to which resting HRV is determined by vagal (parasympathetic) activation. At rest, a major neural influence on heart rate is prefrontally modulated and vagally mediated tonic inhibitory control [37]. During inspiration, vagal or parasympathetic activity is reduced (leading to an increase in heart rate), whereas during expiration vagal activity is enhanced (leading to a decrease in heart rate). Consequently, higher parasympathetic activity leads to more beat-to-beat variability in heart rate. 

Obtained at rest, vagally mediated HRV is a physiological trait marker of emotion regulation capacity, as the vagus nerve is connected to the same neural network (including the prefrontal cortex, amygdala, and hypothalamus) that is also involved in emotion regulation [25,38-40]. In particular, vagally mediated HRV indexes the inhibitory capacity of the prefrontal cortex that is required for regulation [40]. A growing number of empirical studies support the assumption that resting HRV is related to self-regulatory strength and emotion regulation [41-44]. From a developmental perspective, increases in vagal tone during early adolescence are related to fewer self-reported problems with emotion regulation [45]. In contrast, low HRV is related to expected outcomes of emotion regulation deficiency: depression, anxiety, hostility and a tonic negative mood [46,47]. Similarly, patients suffering from bipolar or borderline personality disorder, both characterized by affective instability, are also marked by low vagally mediated HRV [48-50]. Indeed, lower vagal activation (measured as respiratory sinus arrhythmia) has been related to emotional dysregulation in borderline patients, as indicated by more frequent and more intense negative emotions [51]. Combined, these findings suggest that HRV is linked to the capacity to inhibit one’s emotional responses and to avoid excessive emotional responding [25,40]. Consequently, we expect vagally mediated HRV to be negatively related to affective instability.

While most of the studies discussed above focused on negative affective functioning, recent research suggests that high vagally mediated HRV may be particularly associated with positive emotionality [52-54]. Thus, in our study we extend upon previous findings by examining whether vagally mediated HRV is related to instability of both positive and negative affect in daily life.

### Overview of the current study

This study investigated how affective instability is related to self-reported use of emotion regulation strategies and parasympathetically mediated HRV. We measured both positive and negative affective instability using the experience sampling method (ESM) [55], which provided repeated assessments of people’s affective experiences as they naturally occurred in daily life. ESM maximizes ecological validity and overcomes the memory biases associated with retrospective self-reports [56]. We used the MSSD as an index of affective instability [17]. Self-reported use of reappraisal and suppression were assessed on the first day of the study. We measured heart rate at the beginning, middle and end of the study. Vagally mediated HRV was quantified using two measures: a time domain measure and a frequency domain measure (see below for details). We used multilevel modeling to examine how these self-reported and physiological indices of emotion regulation were related to affective instability in daily life. Based on the previously described theoretical and empirical literature, we hypothesized that reappraisal and vagally mediated HRV would be negatively related to affective instability.

## Methods

### Ethics statement

This study was approved by the ethics committee of the Faculty of Psychology and Educational Sciences, KU Leuven and all participants provided written informed consent.

### Participants

One hundred undergraduates were recruited to participate as part of a larger study. Participants were selected from an initial sample of 439 undergraduates to represent a wide range depressive symptom levels, based on pre-screening with the Centre for Epidemiologic Studies Depression scale [57] (CES-D, range = 0 – 50, *M* = 19.27, *SD* = 12.53). Using a stratified sampling approach [58], approximately equal numbers of participants were recruited from the entire CES-D pre-screening range to maximize variability otherwise lacking in a typical student sample. At the time of pre-screening, 55 participants had CES-D scores ≥ 16, a clinical cutoff proposed by Radloff [57], and 32 participants had CES-D scores ≥ 27, a more conservative clinical cut-off proposed by Gotlib, Lewinsohn, and Seeley [59].

One participant withdrew early, four were excluded due to missing ESM data (three participants with equipment malfunction and one participant showing poor compliance with more than 40% missing data) and 12 were excluded due to poor quality heart rate data (see below), leaving a final sample of 83 participants (52 women) ranging in age from 18 to 24 years (*M* = 19.02, *SD* = 1.28). Participants excluded from data analyses did not differ from the rest of the sample on age, sex, depressive symptoms, self-reported use of reappraisal and suppression, and either mean levels or instability of positive and negative affect (all ps > .17). Individuals suffering from cardiovascular disease and/or taking medication affecting the cardiovascular or central nervous systems were excluded during recruitment. The data reported here are drawn from a larger study for which participants were compensated with €70. 

### Materials and procedure

The study lasted for eight consecutive days. On the morning of the first day, participants came to the laboratory for a heart rate measurement, to complete the emotion regulation questionnaire, and to receive instructions and materials for the experience sampling. For the following seven days, participants carried a Palm Tungsten E2 palmtop computer and reported on their feelings in daily life. Participants returned for a second lab session during the experience sampling week, which included another heart rate measurement. On the eighth day, participants attended a final lab session to complete a number of lab tasks, including a third heart rate assessment. Participants then returned their palmtops, were debriefed and financially compensated.

#### Heart rate assessment

To ensure a high trait component in our indices of vagally mediated HRV, we obtained repeatedly measured heart rate in identical settings [60].Three resting heart rate measurements were obtained for each participant: the first and third occurred on Days 1 and 8 of the study, respectively. The second measurement was obtained on Day 4, 5 or 6 of the study, depending on each participant’s availability. This ensured that no two heart rate measurements occurred on consecutive days. During each heart rate assessment, participants were seated in individual cubicles and were instructed to sit quietly and relax. Heart rate (interbeat intervals) was assessed at rest for 10 minutes at a sampling rate of 1000 Hz using a Polar RS800CX (Polar Electro Oy, Kempele, Finland) [61,62]. All participants were asked to refrain from alcohol for 12 hours and from caffeine, smoking and physical exercise for two hours before each measurement. 

#### Self-reported emotion regulation

Participants completed the 10-item Emotion Regulation Questionnaire [24], which consists of a 6-item reappraisal subscale (α = .69) and a 4-item suppression subscale (α = .82). 

#### Experience sampling

Participants received the palmtop (programmed using a modified version of the Experience Sampling Program) [63] to be used as an electronic diary of their feelings for the following week. Throughout the seven days of experience sampling, the palmtop prompted participants to report their feelings 10 times a day according to a stratified random interval scheme (i.e., the day was divided into 10 equal intervals with one prompt programmed to occur randomly within each interval). At each sampling moment, participants were asked to rate their current feelings using six adjectives (happy, relaxed, angry, sad, depressed, anxious) on a scale from 0 to 100 using a continuous slider (0 = *not at all*; 100 = *very*). The specific emotion items were chosen in order to reflect all quadrants of the circumplex model of affect [64]. Positive affect (PA) and negative affect (NA) scales were calculated by averaging the positive and negative items respectively. Multilevel reliability analyses indicated that the NA scale had reliability of .66 and the PA scale had a reliability of .63 (these are equivalent to Cronbach’s alphas in a multilevel context) [65]. Compliance with the experience sampling procedure was very good: on average participants completed 91.27% of scheduled beeps (*SD* = 6.32%, range = 67 - 100%). 

### Data analysis

#### Heart rate variability

Sequential interbeat intervals were downloaded using the software Polar Pro Trainer 5. The following pre-processing and analyses were conducted by the last author to obtain HRV scores for each participant, before the main analyses relating vagally mediated HRV to affective instability were conducted by the two first authors: All signals were visually inspected and 12 participants were discarded from the database due to equipment failure on one or more of the heart rate measurements. HRV analysis was performed using the ARTiiFACT software [66]. First, a five minute period (minutes 3 to 8) was selected from the 10-minute signal to ensure the selection of a period of optimal relaxation and to exclude setting-related disturbances (starting the recording, experimenter leaving or entering the room, etc.). Then, measurement artifacts were identified by applying a criterion threshold that was calculated for each individual based on his/her heart rate and distribution of interbeat interval lengths. This distribution-based definition of thresholds marking measurement artifacts is considered the most appropriate and precise identification algorithm as described by Berntson et al. [67,68]. Erroneous beats were deleted and substituted by means of cubic spline interpolation of neighboring intervals. We extracted two indices of vagally mediated HRV using ARTiiFACT: the root Mean Square Successive Difference (rMSSD), a time domain measure, and the High Frequency component (HF, 0.15-0.40 Hz), a frequency domain measure. Both measures reflect vagally mediated parasympathetic influences on the heart [36,60,69] and are highly correlated [70-73]. However, rMSSD appears to also capture sympathetic activation [71]. For each HRV index, we took the mean of each participant’s three measurement occasions to maximize the proportion of the trait component and minimize state-dependent influences on vagally mediated HRV [60].

#### Preprocessing of affective data

Analyzing MSSD in a multilevel model requires some preprocessing of the data. Our analysis focused on the time series of squared successive differences (denoted as *SSD*_*ij*_ where the first index refers to person *i* and the second to measurement *j*), in line with the recommendations of [17]. The *SSD*_*ij*_’s were calculated separately for positive and negative affect. To remove evening to morning differences, we excluded difference scores indicating mood changes between days. In addition, *SSD*_*ij*_ was log transformed to adjust for skewness of the distribution (the zero values of *SSD*_*ij*_ were replaced with half of smallest non-zero value to allow for log transformation). Skewness values for the SSD before log-transformation were 3.47 and 5.29 for PA and NA, respectively. After log transformation, skewness values decreased to -0.97 and -0.44 for PA and NA, respectively.

#### Statistical model

The nested structure of the data (observations nested within participants) necessitates a multilevel regression approach [74]. Following Jahng et al. [17], we modeled the (log transformed) within-person squared successive differences of affect (*lnSSDAffect*_*ij*_) using a multilevel random intercept model in which the Level 1 random intercept (*β*_*0j*_) was predicted by vagally mediated HRV, reappraisal or suppression at Level 2 (denoted below by the generic symbol *ER*_*j*_ ). We first ran unconditional models with no Level-2 predictors. The fixed effects estimates from the unconditional models represent average levels of (log transformed) instability across the sample. For PA, these were *B* = 4.33, *SE* = 0.07, *p* < .001, and for NA: *B* = 2.42, *SE* = 0.15, *p* < .001. Random effects estimates from these models indicated significant amounts of between-person variability in levels of instability for both PA (*SD* = 0.57, *p* < .001) and NA (*SD* = 1.33, *p* < .001). In our main analyses, we modeled this between-person variability in PA and NA instability as a function of emotion regulation. Thus, the Level 2 slope (*γ*_*01*_) represents the association between each index of emotion regulation and affective instability. PA and NA instability were modeled in separate analyses. The model equations were as follows: 

$$\text{Level-1: }\ln(SSDAffect_{ij})=\beta_{0j}+r_{ij}$$

$$\text{Level-2: }\beta_{0j}=\gamma_{00}+\gamma_{01}*ER_{j}+\mu_{0j}$$

$$\text{where }r_{ij}\text{\textasciitilde{}}(0\text{, }\sigma_{r}^{2})\text{ and }\mu_{0j}\text{\textasciitilde{}}(0\text{, }\sigma_{\mu 0}^{2})$$

When assessing affective fluctuations, several researchers have underlined the importance of controlling for mean levels of affect to resolve the issue of linear dependencies between variability scores and the mean [2,75]. Therefore, in a second step of each model, we controlled for mean level of PA or NA at Level 2, such that the Level 2 equation became:

$$\text{Level-2: }\beta_{0j}=\gamma_{00}+\gamma_{01}*ER_{j}+\gamma_{02}*Mean\;Affect_{j}+\mu_{0j}$$

## Results

Table 1 displays descriptive statistics and Pearson correlations among all measures. Mean levels of PA and NA were negatively correlated, whereas instability of PA and NA were positively associated. NA mean level correlated positively with both PA and NA instability. In contrast, PA mean level was negatively associated with PA and NA instability, although there was only conclusive evidence for the latter (*p* < .05). The two HRV indices were strongly positively correlated, however neither rMSSD nor HF was related to reappraisal or suppression. Finally, self-reported use of reappraisal and suppression were also unrelated.

**Table 1:** Descriptive Statistics and Correlations among all Study Variables.

	**1.**	**2.**	**3.**	**4.**	**5.**	**6.**	**7.**	**Mean**	**SD**
**1. PA mean level**	1.00							57.15	12.77
**2. NA mean level**	-.65**	1.00						16.06	11.00
**3. PA instability**	-.19	.22*	1.00					397.20	202.03
**4. NA instability**	-.40**	.67**	.48**	1.00				139.02	114.43
**5. rMSSD (HRV)**	.02	.03	-.31**	-.15	1.00			38.41	15.47
**6. HF**	.05	.04	-.30**	-.16	.86**	1.00		633.77	542.36
**7. Reappraisal**	.02	-.04	-.03	-.04	-.14	-.08	1.00	4.39	0.84
**8. Suppression**	.01	.03	.14	.04	-.02	-.11	.03	3.03	1.29

Note. *N* = 83. Means and *SD*s for PA and NA instability are based on raw mean square successive differences rather than log transformed values. PA = positive affect; NA = negative affect; rMSSD = root mean square successive difference; HF = high frequency component expressed in absolute units; HRV = heart rate variability.

* *p* < .05, ** *p* < .01.

Our main analyses consisted of eight multilevel models predicting PA or NA instability from each measure of emotion regulation (see model equations above). 

### Associations between emotion regulation and PA instability

**Figure 1:**
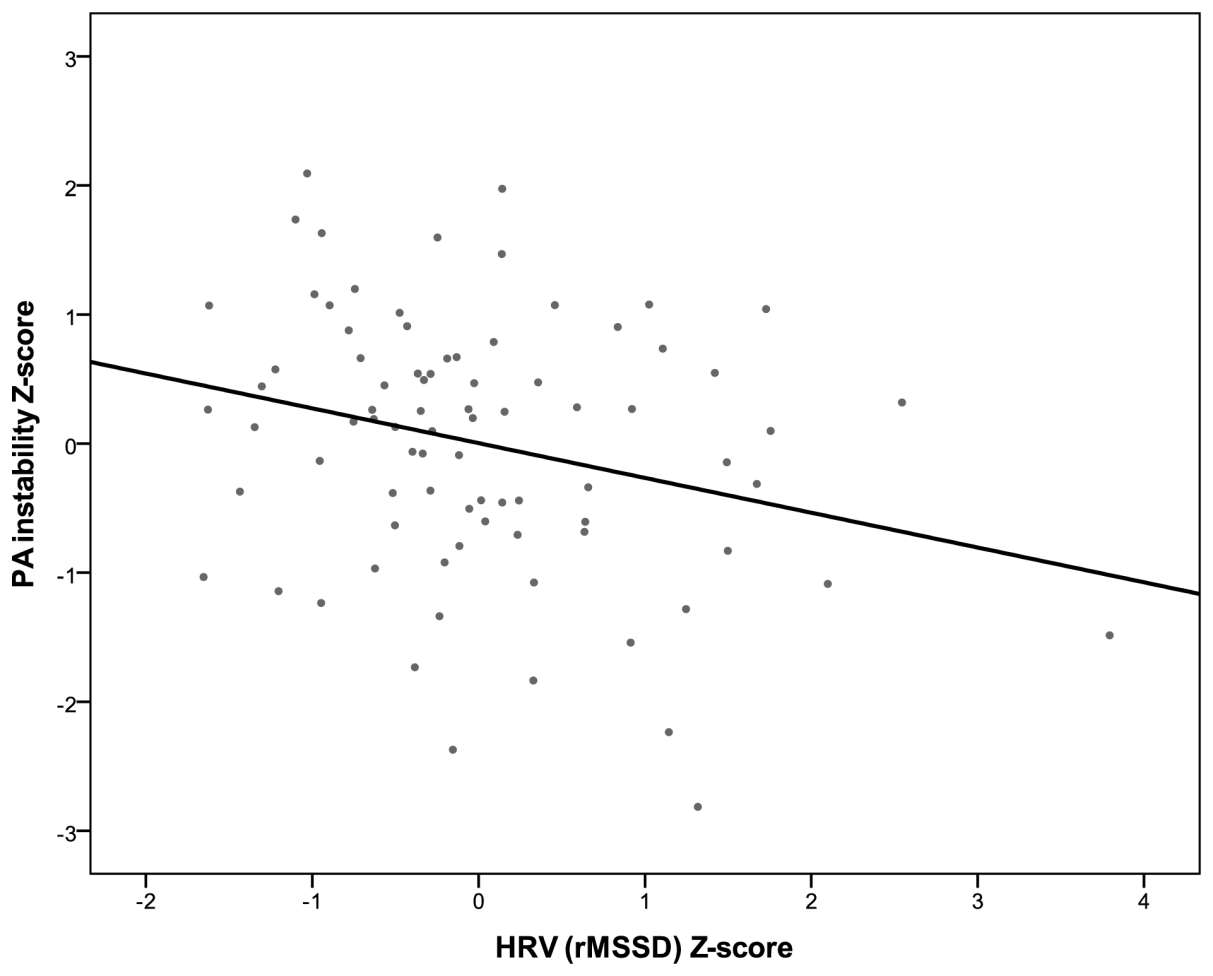
Standardized PA instability scores plotted against standardized HRV (rMSSD) values. Individual PA instability values are person-specific intercepts obtained from an intercept-only multilevel model with log-transformed PA squared successive differences as the outcome. Linear fit line shown is based on all observations.

**Figure 2:**
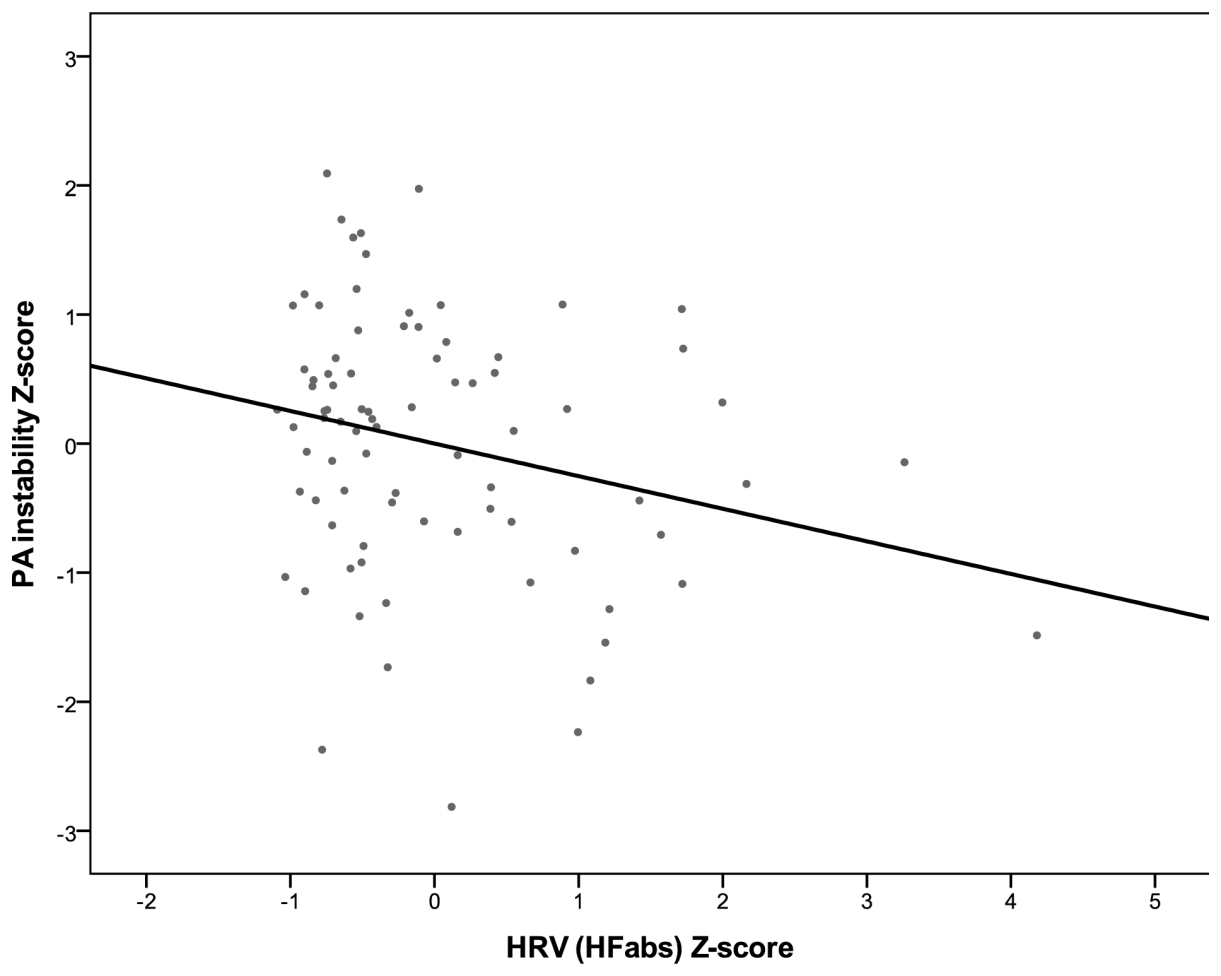
Standardized PA instability scores plotted against standardized HRV (HFabs) values. Individual PA instability values are person-specific intercepts obtained from an intercept-only multilevel model with log-transformed PA squared successive differences as the outcome. Linear fit line shown is based on all observations.

Models 1 and 2 examined how PA instability was related to self-reported reappraisal and suppression, respectively. As shown in Table 2, neither reappraisal nor suppression were related to PA instability. After controlling for mean levels of PA at Step 2 of each model, there was no evidence for associations between PA instability and either reappraisal or suppression (see Table 2). In Models 3 and 4, we examined the associations between each index of vagally mediated HRV and PA instability. In contrast to results for the self-reported emotion regulation, there was conclusive evidence that both the rMSSD and HF indices of HRV were negatively associated with PA instability (see Table 2). This evidence remained after controlling for mean level of PA (see Step 2 in Models 3 and 4). Figures 1 and 2 plot the associations between PA instability with the rMSSD and HF indices of vagally mediated HRV, respectively. Figure 1 shows that one participant had an rMSSD value more than 3 *SD*s above the sample mean and reference values representative for the general propulation [76] and can therefore be considered an outlier. Similarly, Figure 2 shows that two participants were outliers on the HF index. To ensure that our results were not unduly influenced by these outliers, we repeated Model 3 excluding the individual with an extreme score on rMSSD: There was still evidence, although somewhat weaker, for an association between rMSSD and PA instability both before (β = -0.16, *SE* = 0.08, *p* = .049) and after controlling for PA mean level (β = -0.16, *SE* = 0.08, *p* = .044). Similarly, we re-ran Model 4 excluding the two participants who were outliers on HF: Again, there was still evidence, although somewhat weaker, for an association between HF and PA instability both before (β = -0.17, *SE* = 0.08, *p* = .045) and after controlling for PA mean level (β = -0.16, *SE* = 0.09, *p* = .061).

**Table 2:** Results of Multilevel Models Predicting Instability (MSSD) of Positive Affect from Self-Reported and Physiological Indices of Emotion Regulation.

		**Step 1**		**Step 2**	
**Predictor**	**Parameter**	***B* (SE)**	***p***	***B* (SE)**	***p***
**Model 1**					
Intercept (PA MSSD)	*γ_00_*	4.33 (0.07)	< .001	4.32 (0.07)	< .001
Reappraisal	*γ_01_*	-0.02 (0.07)	.753	-0.02 (0.07)	.790
PA mean level	*γ_02_*	―	―	-0.14 (0.09)	.134
**Model 2**					
Intercept (PA MSSD)	*γ_00_*	4.32 (0.07)	< .001	4.32 (0.07)	< .001
Suppression	*γ_01_*	0.04 (0.06)	.475	0.04 (0.06)	.461
PA mean level	*γ_02_*	―	―	-0.14 (0.09)	.136
**Model 3**					
Intercept (PA MSSD)	*γ_00_*	4.33 (0.07)	< .001	4.33 (0.07)	< .001
rMSSD (HRV)	*γ_01_*	-0.18 (0.07)	.010	-0.18 (0.06)	.009
PA mean level	*γ_02_*	―	―	-0.13 (0.09)	.121
**Model 4**					
Intercept (PA MSSD)	*γ_00_*	4.32 (0.07)	< .001	4.32 (0.07)	< .001
HF	*γ_01_*	-0.17 (0.06)	.006	-0.16 (0.06)	.008
PA mean level	*γ_02_*	―	―	-0.13 (0.09)	.144

In Step 2 mean affect was controlled for.

### Associations between emotion regulation and NA instability

 Models 5 and 6 examined associations between NA instability and self-reported reappraisal and suppression, respectively. Similar to the results for PA, there was no evidence that NA instability was associated with self-reported reappraisal or suppression either before or after controlling for NA mean level (see Models 5 and 6 in Table 3). Finally, Models 7 and 8 tested associations between each index of vagally mediated HRV and NA instability. We found no evidence for an association between NA instability and either rMSSD or HF at Step 1 of Models 7 and 8. However, after controlling for mean NA (Step 2), there was some evidence for an association between each index of vagally mediated HRV and NA instability (see Table 3). When we repeated Models 7 and 8 excluding the outliers on each index of vagally mediated HRV (as defined above), these results replicated: no (or weak) evidence that either rMSSD (β = -0.18, *SE* = 0.18, *p* = .308) or HF (β = -0.37, *SE* = 0.19, *p* = .061) were related to NA instability before controlling for mean level of NA. However, after controlling for NA mean level, there was evidence for both associations (rMSSD: β = -0.25, *SE* = 0.10, *p* = .017; HF: β = -0.32, *SE* = 0.12, *p* = .009).

**Table 3:** Results of Multilevel Models Predicting Instability (MSSD) of Negative Affect from Self-Reported and Physiological Indices of Emotion Regulation.

		**Step 1**		**Step 2**	
**Predictor**	**Parameter**	***B* (SE)^a^**	***p***	***B* (SE)^b^**	***p***
**Model 5**					
Intercept (NA MSSD)	*γ_00_*	2.42 (0.15)	< .001	2.42 (0.09)	< .001
Reappraisal	*γ_01_*	-0.02 (0.15)	.907	0.03 (0.11)	.807
NA mean level	*γ_02_*	―	―	1.08 (0.15)	< .001
**Model 6**					
Intercept (NA MSSD)	*γ_00_*	2.42 (0.15)	< .001	2.42 (0.09)	< .001
Suppression	*γ_01_*	0.17 (0.14)	.225	0.14 (0.09)	.137
NA mean level	*γ_02_*	―	―	1.08 (0.14)	< .001
**Model 7**					
Intercept (NA MSSD)	*γ_00_*	2.43 (0.15)	< .001	2.42 (0.09)	< .001
rMSSD (HRV)	*γ_01_*	-0.18 (0.15)	.221	-0.21 (0.09)	.020
NA mean level	*γ_02_*	―	―	1.09 (0.14)	< .001
**Model 8**					
Intercept (NA MSSD)	*γ_00_*	2.42 (0.15)	< .001	2.42 (0.09)	< .001
HF	*γ_01_*	-0.25 (0.14)	.080	-0.21 (0.10)	.035
NA mean level	*γ_02_*	―	―	1.07 (0.14)	< .001

In Step 2 mean affect was controlled for.

Note. Level 2 predictors were standardized. MSSD = mean square successive difference; NA = negative affect; rMSSD = root mean square successive difference; HF = high frequency component; HRV = heart rate variability.

^a^ *d.f.* = 81. ^b^ d.f .= 80.

### Ancillary analyses relating other measures of affect dynamics

We also examined how emotion regulation was related to two other measures of affect dynamics, the within-person standard deviation and autocorrelation, which are sub-components of affective instability [17]. We found no statistical evidence for associations between self-reported reappraisal or suppression and either the standard deviation or autocorrelation of either PA or NA (rs < |.15|, ps > .18). Similarly, there was no evidence that either of the two HRV indices was related to the autocorrelation of PA or NA, or to the standard deviation of NA (rs < |.13|, ps > .26). However, there was some weak evidence that the two HRV indices were negatively associated with the standard deviation of PA (rMSSD: *r* = .23, *p* = .04; HF: *r* = .21, *p* = .06).

## Discussion

This study investigated how affective instability in daily life is related to self-reported emotion regulation (reappraisal and suppression) and to a physiological indicator of emotion regulation capacity, namely vagally mediated HRV. We found that neither self-reported use of reappraisal nor self-reported use of suppression were related to affective instability. In contrast, higher HRV, as indexed by both the rMSSD and HF measures, was associated with lower levels of affective instability, particularly of positive affect. When controlling for mean levels of affect, we also found a negative relationship between vagally mediated HRV and instability of negative affect. By controlling for mean levels of affect, the between-person variability in tonic affect level is removed. Therefore the relationship between vagally mediated HRV and affective instability cannot be attributed to tonic mood differences. As previous research [46,47,53] has shown that vagally mediated HRV is related to such mean levels of affect, it may be important to control for individual mood differences when examining variability. Nevertheless, the substantive interpretation of analyses controlling for mean levels of affect is less straightforward. We therefore advise caution when interpreting the results regarding instability of NA.

Our failure to find a relationship between self-reported use of emotion regulation and affective instability might be due to how reappraisal and suppression were assessed in the current study. First, self-report measures are prone to memory and social desirability biases. Second, the ERQ assesses the extent to which people habitually use reappraisal and suppression, but not necessarily their effectiveness in regulating mood [24,77]. In line with this, we found that self-reported use of reappraisal and suppression were unrelated to vagally mediated HRV, underscoring the importance of distinguishing emotion regulation use from emotion regulation success [77].

In contrast to self-reported use of emotion regulation, vagally mediated HRV captures a basic prerequisite for effective emotion regulation, as it indexes the inhibitory capacity of the prefrontal cortex [40,78]. Vagally mediated HRV can therefore be seen as an indicator of how well people are actually capable of regulating their emotions. It makes sense that one’s capacity to effectively regulate one’s emotions is more strongly related to one’s level of affective instability than the specific types of emotion regulation strategies one uses. Moreover, our finding that vagally mediated HRV is particularly related to lower PA instability, suggests that vagally mediated HRV may be especially important in the regulation of positive affective states. In line with this finding, recent research [52,53] has shown that vagally mediated HRV is related to tonic positive emotionality and to the experience of more enduring positive emotions. Taken together with the current study’s findings, vagally mediated HRV appears to relate to both the mean level and temporal dynamics of PA. 

It is intriguing to note that “instability” at the physiological level relates to stability at the experiential level. While one of the vagally mediated HRV indices we used (rMSSD) is essentially the same statistical index as the measure of affective instability (MSSD), these two measures are on markedly different time scales (heart rate was assessed continuously and changes over the course of seconds whereas we assessed affective changes over the course of hours). In other words, our finding may suggest that short-term physiological variability may be required to assure longer-term stability in affect, much like quick small adjustments are needed to maintain a longer-term upright posture [79]. This points to the possibility that dynamics at different time scales and modalities may mutually influence each other [80].

We note that not all research points to the conclusion that affective instability is maladaptive. Emotional flexibility, defined as the ability to be emotionally responsive to changing environmental stimuli, has been proposed as a hallmark of psychological health [4] and several studies have found supporting evidence for this [5,81]. However, in order to infer affective inflexibility it is crucial to assess how people’s responses map onto environmental demands and therefore the objective situation needs to be taken into account. Given that the current study did not control for situational factors, our findings cannot easily be interpreted in terms of affective (in)flexibility. Furthermore, we found a relationship between affective instability and vagally mediated HRV primarily for PA, whereas most research supporting the notion that affective inflexibility is maladaptive is specific to NA. In general, more research is needed to dissect the precise relationships between inflexibility–as, for example, measured using autocorrelation [5]–and instability, as measured using MSSD [82].

The current study is not without limitations. First, due to the cross-sectional design of our study, it is not possible to determine the direction of causality in the relationship observed between HRV and affective instability. However, resting HRV is generally considered to be a disposition: it displays high test-retest reliability [83] and heritability [84]. Vagally mediated HRV is therefore likely to precede affective instability in the causal chain. However, to establish a causal relationship, future studies could for instance manipulate resting HRV by physical training and investigate its impact on affective instability [85]. Second, we cannot exclude the possibility that our choice of time-window (mins 3 to 8 of a 10 min heart rate recording) was too early to ensure that all participants, including those with less emotion regulation capacity, reached the same degree of relaxation. However, we consider this risk as rather low, given the fact that our repeated measures make nervousness in the laboratory setting less likely in the second and third sessions.

To our knowledge there is no research that has directly investigated how vagally mediated short-term HRV as a trait-marker for emotion regulation capacity relates to affective instability in daily life. Taken together, the current findings show that individuals with high vagally mediated HRV are less prone to sudden and large fluctuations in their positive mood. This suggests that the capacity to effectively regulate emotions, as reflected by parasympathetically mediated HRV, may be a potential protective factor against the instability of positive affect in daily life.
